# Predictors and Impact of Cardiogenic Shock in Oldest-Old ST-Elevation Myocardial Infarction Patients

**DOI:** 10.3390/jcm14020504

**Published:** 2025-01-14

**Authors:** Luca Donazzan, Alessandro Ruzzarin, Simone Muraglia, Enrico Fabris, Monica Verdoia, Filippo Zilio, Giorgio Caretta, Andrea Pezzato, Gianluca Campo, Matthias Unterhuber

**Affiliations:** 1 Department of Cardiology, San Maurizio Hospital, 39100 Bolzano, Italy; 2 Department of Cardiology, Santa Chiara Hospital, 38122 Trento, Italy; 3 Cardiothoracovascular Department, University of Trieste, 34149 Trieste, Italy; 4 Department of Cardiology, Ospedale degli Infermi, ASL Biella, 13875 Biella, Italy; 5 Cardiology Unit, Sant’Andrea Hospital, ASL 5 Liguria, 19121 La Spezia, Italy; 6 Cardiology Unit, Azienda Ospedaliera-Universitaria di Ferrara, 44124 Cona, Italy

**Keywords:** elderly, STEMI, cardiogenic shock, oldest old

## Abstract

**Background**: Cardiogenic shock (CS) is the most frequent cause of in-hospital mortality after ST-elevation myocardial infarction (STEMI). Data about CS in very elderly (age ≥ 85 years) STEMI patients are scarce. We sought to assess the prognostic factors and the short- and mid-term impact of CS in this population. **Methods**: Consecutive very elderly STEMI patients undergoing invasive treatment were included in a retrospective multicenter registry. **Results**: Among 608 patients, 72 (11.8%) fulfilled experienced CS. Peripheral artery disease (PAD) (OR: 2.25, 95% CI: 1.29–3.92, *p* < 0.01) and cardiac arrest at presentation (OR: 4.36, 95% CI: 2.32–8.21, *p* < 0.01) were the major independent predictors of CS. Age (HR: 1.07, 95% CI: 1.03–1.11, *p* < 0.001), PAD (HR: 1.29, 95% CI: 1.01–1.66, *p* = 0.045), previous MI (HR: 2.16, 95% CI: 1.32–3.55, *p* = 0.002), and cardiac arrest at presentation (HR: 1.59, 95% CI: 1.29–1.96, *p* < 0.001) were the major independent predictors of death. CS was associated with a higher risk of mortality at 30 days (adjusted HR: 4.21, 95% CI: 2.19 to 7.78, *p* < 0.01) mostly driven by higher intraprocedural and in-hospital mortality. Among patients who survived the acute phase and hospitalization, CS at presentation was not associated with a higher mortality risk during the remaining follow-up period (log-rank *p* = 0.78). **Conclusions**: At short-term follow-up, very elderly STEMI patients presenting with CS had a higher risk of mortality when compared to non-CS patients. Interestingly, CS patients surviving the acute phase showed a similar survival rate to non-CS patients after discharge.

## 1. Introduction

The average age of the population is growing and consequently that of cardiologic patients. Urgent coronary revascularization with primary percutaneous coronary intervention (pPCI) in the setting of ST-elevation acute myocardial infarction (STEMI) has been shown to improve mortality also in this fragile population [1]. One of the most feared acute complications of STEMI is cardiogenic shock (CS), which is a complex multifactorial condition associated with high morbidity and very high short-term mortality [2]. The paucity of data regarding the oldest-old (i.e., age ≥ 85 years old) STEMI patients undergoing invasive management in the setting of CS hampers the interpretation of data in this ominous condition. As a matter of fact, elderly patients presenting with CS have age as an important and unmodifiable prognostic factor. Age itself is in fact associated with higher in-hospital mortality [3]. This characteristic has also become a highly relevant factor in the decision-making process as a guide to the use of invasive therapies. As precise data on the incidence and predictors of CS in the oldest-old STEMI patients are scarce, its relationship with mortality after invasive management is unclear. To address this gap in knowledge, we evaluated the incidence of CS-complicating STEMI in the oldest-old patients undergoing an invasive treatment strategy (either coronary angiography only or PCI) and assessed its impact on mortality in the mid-term period.

## 2. Materials and Methods

### 2.1. Study Population and Design

A retrospective analysis was performed on very elderly STEMI patients (age ≥ 85 years old) undergoing coronary angiography and included in a multicenter registry of six Hub hospitals (i.e., Biella, Bolzano, Ferrara, Trento, Trieste, and La Spezia) in Northern Italy between January 2010 and August 2023. Patients were enrolled in a registry with detailed baseline clinical, time to treatment, laboratory, angiographic, and follow-up data. Exclusion criteria were age < 85 years old, conservative treatment strategy (i.e., medical therapy without coronary angiography or PCI), and missing data. Indications for pPCI were in accordance with international guidelines [1] and institutional protocols. All the surviving patients were followed up for 12 months with regular outpatient clinic visits or telephone interviews.

### 2.2. Definitions

STEMI criteria were defined according to the fourth universal definition of Myocardial Infarction [4]. STEMI-related CS has been defined according to Killip definition, i.e., Killip class IV, an event with systolic blood pressure less than 90 mmHg for greater than 30 min, not responsive to fluid resuscitation alone, and felt to be secondary to cardiac dysfunction [5]. Intraprocedural complications were considered as bleeding according to Bleeding Academic Research Consortium (BARC) criteria, acute kidney injury (AKI), or intraprocedural death. In-hospital complications were considered as target vessel failure with or without revascularization, stent thrombosis, stroke, bleeding according to Bleeding Academic Research Consortium (BARC) criteria, heart failure, and relapsing angina.

### 2.3. Main Outcome

The main outcome of the study was to evaluate the predictors and impact of cardiogenic shock in the oldest-old STEMI patients. The secondary outcome was to identify the incidence of in-hospital and out-of-hospital mortality in patients presenting with CS complicating a STEMI (STEMI-CS).

### 2.4. Statistical Analysis

All analyses were performed using SPSS Statistics Software for Mac (Version 29, Armonk, NY, USA: IBM Corp). Patients were grouped according to CS diagnosis. Absolute frequencies and percentages were used for categorical variables. Continuous variables are presented as mean and standard deviation. The normal distribution of continuous variables was tested using the Kolmogorov–Smirnov test. Categorical variables were reported as frequencies and percentages and compared by means of the X^2^ or Fisher test, as appropriate. Continuous variables were visually assessed for distribution and subsequently reported as mean (SD) or median (quartile 1–quartile 3 [Q1–Q3]) and compared using the Student’s *t* test or Wilcoxon–Mann–Whitney U test, as appropriate. Binary logistic regressions were run to obtain the predictors of CS. To adjust for covariates, significant univariate predictors were entered into a multivariable model. Odds ratios (ORs) and associated 95% CIs were derived, and Cox regression models presented with hazard ratios (HRs) and associated 95% CIs. The cumulative unadjusted frequencies of all-cause mortality at different step points were obtained with the Kaplan–Meier method and compared through the log-rank test. Two-tailed *p* < 0.05 were considered statistically significant.

## 3. Results

The retrospective evaluation of the cath-lab registries allowed a preliminary inclusion of 674 oldest-old STEMI patients. The following criteria were used for the 66 excluded patients: coronary angiography not performed and incomplete baseline data. Six hundred and eight oldest-old STEMI patients were included in the final analysis.

Seventy-two (11.8%) patients fulfilled the criteria for CS. The clinical characteristics of the population are reported in Table 1. Female prevalence in oldest-old STEMI patients showed a trend in CS when compared to male patients (61.1 vs. 49.3%, *p* = 0.06). Oldest-old STEMI patients presenting with CS had more often peripheral artery disease (PAD) (40.0 vs. 23.4%, *p* < 0.01) and previous MI (25.7 vs. 13.3%, *p* < 0.01). Cardiac arrest was significantly more common (29.2 vs. 8.0%, *p* < 0.01) in the CS group compared with the non-CS group at presentation (Table 1).

Baseline platelet count and hemoglobin were not statistically different in the two groups. Creatinine was significantly higher in patients with CS as reported in Table 2.

There was no clear association between the culprit lesion and CS, but when the culprit lesion was a bypass, the arterial artery bypass was significantly more associated with CS than the non-CS group (2.8 vs. 0.4%, *p* = 0.03). The femoral artery was the most often used vascular access for coronary angiography (CAG) in patients with CS (41.7% vs. 22.6%, *p* < 0.01). Inotropic support and mechanical circulatory support were significantly more adopted in oldest-old STEMI patients presenting with CS (76.4 vs. 4.9%, *p* < 0.01 and 13.9 vs. 0.6%, *p* < 0.01). Contrast media volume was comparable between the two groups (165.0 ± 75.9 vs. 167.5 ± 80.4 mL, *p* = 0.81), but contrast-associated AKI after CAG was significantly higher in patients presenting with CS (50.0 vs. 27.4%, *p* < 0.01). Further procedural characteristics are reported in Table 3.

CS patients had more often (78% vs. 63%, *p* = 0.014) thrombolysis in myocardial infarction (TIMI) flow 0 before pPCI, and a poorer (63% vs. 81%, *p* < 0.001) procedural success (intended as TIMI flow 3 restoration post-PCI) than those without CS. TIMI flow improvement post-PCI was higher in patients with CS than in those without (82% vs. 70%, *p* = 0.047). Manual thrombectomy was performed in 33% of the CS patients and 23% of the non-CS patients (*p* = 0.064).

Compared with the non-CS group, intraprocedural complications, intraprocedural mortality, and in-hospital mortality of patients presenting with CS complicating STEMI were significantly higher (45.8 vs. 26.5%, 15.3 vs. 1.7%and 44.4 vs. 11.8%, respectively, *p* < 0.01 for all) (Table 4). CS was a predictor of intraprocedural complications (HR 3.36, 95% CI: 2.29–4.93, *p* < 0.001).

There was no difference among complications during hospitalization except for heart failure (HF), which was significantly more frequent (*p* < 0.001) in patients who presented with CS (Supplementary Table S1).

There were only two drugs given differently in the two groups at discharge: diuretics and beta-blockers. Diuretics were most frequently prescribed to patients who experienced CS, whereas beta-blockers were mostly prescribed to the non-CS group. Therapy at discharge is reported in Supplementary Table S2.

**Figure 1:**
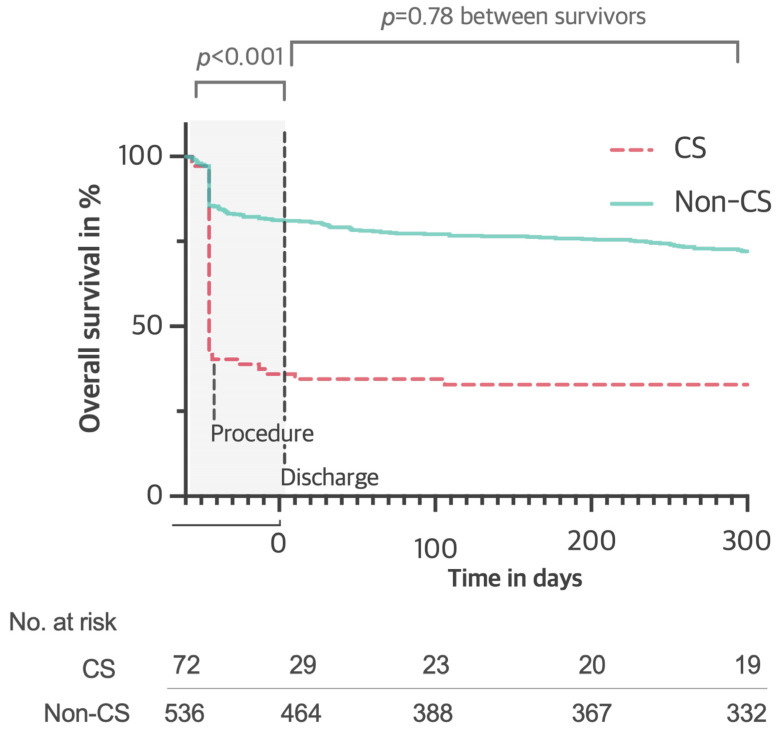
Kaplan–Meyer curves of survival rates.

After discharge, mortality rates were not significantly different at one month, between 1 and 6 months, and between 6 and 12 months between the CS and non-CS groups (*p* = 0.87, *p* = 0.14, and *p* = 0.24, respectively, Figure 1).

CS was associated with a higher risk of mortality at 30 days (adjusted HR: 4.21, 95% CI: 2.19 to 7.78, *p* < 0.01) mostly driven by higher intraprocedural and in-hospital mortality. Among patients who survived hospitalization, CS at presentation was not associated with a higher mortality risk during remaining the follow-up period (log-rank *p* = 0.78).

Peripheral artery disease (OR: 2.25, 95% CI: 1.29–3.92, *p* < 0.01) and cardiac arrest at presentation (OR: 4.36, 95% CI: 2.32–8.21, *p* < 0.01) were predictors of CS.

In the multivariable analysis, age (HR: 1.07, 95% CI: 1.03–1.11, *p* < 0.001), PAD (HR: 1.29, 95% CI: 1.01–1.66, *p* = 0.045), previous MI (HR: 2.16, 95% CI: 1.32–3.55, *p* = 0.002), and cardiac arrest at presentation (HR: 1.59, 95% CI: 1.29–1.96, *p* < 0.001) were the major independent predictors associated with a higher incidence of death. Complete univariate and multivariable analyses are presented in Table 5 and Table 6.

## 4. Discussion

Elderly patients are a heterogeneous population with long-lasting risk factors modifying the cardiovascular structure, adding to frailty and possible drug-related adverse effects. All these characteristics make the elderly more susceptible to STEMI complications. Among them, the most feared is CS. Data from a European cohort revealed a significantly higher prevalence of CS complicating acute MI (AMICS) among patients ≥ 75 years of age compared with younger patients in both STEMI and non-STEMI [6]. In the present study, a cut-off of 85 years was used to define the oldest old, which is considerably higher than the limit of 75 years that is usually set in studies and registries. This poses the present cohort at a higher risk for CS and may in part explain the slightly higher incidence of CS [3].

Interestingly, no gender differences were observed in population prevalence (with a trend shown in 61% females vs. 49%), and neither did female or male genders represent a significant independent predictor for mortality or CS. Previously published papers regarding gender differences in CS showed discrepancies in prevalence [7,8], although not including only STEMI patients, younger patients, and not all with intervention. Other works [9] highlighted disparities between the genders in CS workup. In our cohort, all patients were treated equally, were older compared to previous publications, and were of a well-selected STEMI cohort. Therefore, this paper adds important knowledge to STEMI patients, showing that the outcomes in our well-selected cohort did not differ between genders.

Despite the similar distribution of the common cardiovascular risk factors, a significantly higher proportion of the STEMI-CS population presented with a history of previous myocardial infarction (*p* < 0.01, Table 1). This finding is important, as it highlights the fact that patients who suffer an acute coronary syndrome (ACS) often are on long-term medical treatment [1], especially with beta-blockers, angiotensin-converting enzyme inhibitors, or receptor blockers. In the long term, this medication is given in the attempt to reverse cardiac remodeling [10,11,12] and to increase life expectancy [13,14,15], but their negative inotropic, lusio-, and dromotropic as well as vasodilatative effects [16] may dramatically influence the outcome during the acute context of AMICS by hindering the effect of inotropes and the response to catecholamines. Moreover, left ventricular systolic function can rapidly deteriorate during STEMI, causing AMICS. This is particularly true if the ejection fraction was reduced by a previous ACS. We also investigated whether the so-called “smoker’s paradox” reported in some STEMI patients [17,18] could be reflected in our population. We found that smoke was not associated with higher mortality (HR 0.98; 95% CI: 0.80–1.21, *p* = 0.9). This was also true in a sub-study of the IABP SHOCK II trial and registry [19]. In that analysis, smoke was not predictive of outcomes in patients with CS-complicating AMI. Smokers had similar ejection fractions and were treated similarly to non-smokers, but they were younger and affected by fewer comorbidities, which can partially explain the survival benefit they had before adjustment for important confounders using Cox regression analysis and in the other STEMI populations. Given the advanced age, this cannot be applied to our population.

We found a significantly higher baseline creatinine in the CS group. The reason is probably in the pathophysiological mechanism of CS, with the decrease in cardiac output and consequent end-organ perfusion and/or increase in central venous pressure. CS and acute kidney injury can present as single or combined entities; the cardiorenal syndrome describes this interaction very well. Type-1 cardiorenal syndrome is secondary to a rapid decline in renal perfusion, given mostly by CS. Intraprocedural complications including BARC bleedings and acute kidney injury were significantly higher after revascularization in patients with STEMI-CS, highlighting the frailty of this subgroup and the consequence of procedural challenges and low cardiac output in CS (*p* < 0.01) [20,21].

In this study, PAD was more prevalent in STEMI-CS patients and had been recognized as an independent risk factor of poor outcome (*p* < 0.01). PAD is seen as the expression of a diffuse vascular disease [22] and is frequently associated with coronary artery disease, chronic kidney disease, and previous stroke because they share the same risk factors. The association of two or more of these conditions has a summative effect on the risk of cardiovascular events and worse prognosis [23]. In the context of ACS, patients with PAD are more prone to develop a systolic dysfunction and present more frequently a worse clinical status than those without [24]. Mihatov et al. demonstrated that among patients presenting with AMICS, PAD was associated with worse limb outcomes and survival [25]. PAD was also associated with lower mechanical circulatory support (MCS) utilization rates and those with PAD who received MCS had increased mortality, lower extremity revascularization, and amputation rates. We found that patients with STEMI-CS had more frequent PAD in combination with previous MI history, which indicates a diffuse cardiovascular pathology and a sicker condition in this population. We found that age, PAD, previous MI, and cardiac arrest at presentation were all independent predictors of death in this oldest-old cohort (*p* < 0.01 for all). Furthermore, PAD and cardiac arrest at presentation were independent predictors of CS (*p* < 0.01 for both).

There was no difference among complications during hospitalization except for HF, which was significantly higher in patients who presented with CS. CS is the extreme condition of HF, and once the critical initial phase is passed, there could be various evolutions: stabilization or relapsing episodes of congestion. Interestingly, the analysis of a nationwide cohort of Norwegian STEMI patients [26] shows that HF episodes were more frequent in elderly patients compared to younger and stabilized after the first six months since discharge, which can partially explain our results of similar one-year mortality in the two groups.

Other possible explanations for the higher intraprocedural and in-hospital death of CS patients are as follows: (a) the intrinsic high mortality of CS itself, leading more often to patient’s death during the attempt of revascularization, (b) the principally high age of the current cohort, which is a proven independent predictor of poor outcome in CS [27,28], and (c) by higher need for femoral artery access (*p* < 0.01), inotropic support (*p* < 0.01), and the need of MCS (*p* < 0.01). STEMI-CS was shown to be an independent predictor of procedural complications (HR 3.36, 95% CI: 2.29–4.93, *p* < 0.001) and death (HR 2.08, 95% CI: 1.53–2.84, *p* < 0.001).

Interestingly, STEMI-CS survivors showed a similar event-free survival rate in comparison to the non-CS group after discharge (log-rank *p* = 0.78, Figure 1). This shows that there is potential for a favorable survival expectancy for CS oldest-old patients once the acute event is survived.

There were only two drugs given differently in the two groups at discharge: diuretics and beta-blockers. Diuretics were most frequently prescribed to patients who experienced CS. We expected this result because diuretics relieve symptoms and congestion in HF patients (without improving mortality), and as we observed in our population, CS patients suffered more frequently from HF during hospitalization. Non-CS patients most frequently received beta-blockers at discharge. Beta-blockers are widely demonstrated to be helpful for patients with ejection fraction (EF) < 40% after AMI [29], but their use in patients with EF > 40% is still under debate [14,15]. Given this, we expected a larger use of beta-blockers in the CS group than in the non-CS. Unfortunately, we do not have complete data on EF at discharge, so speculation on the possible explanations of the different uses in the two populations is not possible. The most important effect of beta-blockers is mortality reduction over time, but we did not observe it in our population: Mortality rates were similar in the two groups after discharge. There are two possible reasons for that: The majority of patients in both groups had an EF > 40%, or beta-blocker therapy was added during the visits after discharge.

The findings of the present study can be summarized as follows: (a) To our knowledge, this is the first study that includes ≥85-year-old people with STEMI and explores the different outcomes between STEMI-CS and STEMI at presentation; (b) CS is known to pose a higher risk of mortality, and the oldest-old patients suffer of an even higher risk of CS in STEMI. Age and comorbidities are undoubtedly predictors of CS and higher mortality, but this study shed light on more specific pathologies like PAD, previous MI, and cardiac arrest at presentation. This leads to a lower intraprocedural success with a higher complication rate, burdening even further the in-hospital stay and increasing mortality of frail and oldest-old patients.

Finally, once STEMI-CS patients survived, they showed a similar event-free survival rate compared to non-CS patients. These findings should raise awareness about the possible outcomes in the short term of this patient cohort, not necessarily induce more conservative management in oldest-old STEMI patients complicated with CS due to the perception of a short life expectancy.

### Limitations

Recent evidence suggests that CS is a continuum ranging from pre-shock to refractory shock states and uses additional criteria that go beyond hypotension alone to accurately reflect the severity of illness. Using the Killip classification to define the state of CS is an important limitation, which is mainly driven by the retrospective analysis of a registry that involves patients since 2010. Thus, the lactate level was not available for most of the patients. Recording patient data over several decades implies having a heterogeneous treatment of STEMI and its complications with important consequences on their outcome.

The retrospective nature of this analysis is also a limitation. In fact, without a prospectively designed study, many treatment strategies were at the operator’s or at center’s discretion. Despite the European Society of Cardiology guidelines not advising against urgent revascularization of elderly STEMI patients, this could have been considered by the majority of treating physicians as expensive, risky, and futile. The same must be said for MCS.

Only patients who arrived in CS alive at the Hub hospitals and underwent pPCI were included in this registry. All patients who died before arriving at the hospital or who arrived at the hospital in such serious conditions that it was decided to perform only drug therapy were therefore excluded.

Details on MCS type and MCS duration are missing.

## 5. Conclusions

Cardiogenic shock is an ominous complication of ACS and is more frequent in patients with a history of previous MI or diffuse cardiovascular disease. Very elderly STEMI patients presenting with CS had a higher risk of intraprocedural and in-hospital death leading to a higher mortality at one-month follow-up. Among survivors of hospitalization, this risk seems not to remain during the first year after discharge.

## Figures and Tables

**Table 1:** Clinical characteristics.

Characteristics	STEMI Population (n = 608)	Cardiogenic Shock (n = 72, 11.8%)	Non-Cardiogenic Shock (n = 536, 88.2%)	*p*-Value
Age (years)	88.6 (2.8)	88.9 (3.4)	88.5 (2.7)	0.33
Female sex	308/608 (50.7%)	44/72 (61.1%)	264/536 (49.3%)	0.06
BMI, (kg/m^2^)	24.7 (3.4)	24.8 (3.1)	24.7 (3.4)	0.86
Hypertension	474/606 (78.2%)	57/71 (80.3%)	417/535 (77.9%)	0.65
Dyslipidemia	236/605 (39.0%)	29/70 (41.4%)	207/535 (38.7%)	0.64
COPD	70/605 (11.6%)	8/70 (11.4%)	62/535 (11.6%)	0.95
Diabetes mellitus	129/605 (21.3%)	13/70 (18.6%)	116/535 (21.7%)	0.55
Cerebrovascular disease	62/605 (10.2%)	11/70 (15.7%)	51/535 (9.5%)	0.11
Peripheral artery disease	153/605 (25.0%)	28/70 (40.0%)	125/535 (23.4%)	<0.01
Previous MI	89/605 (14.7%)	18/70 (25.7%)	71/535 (13.3%)	<0.01
Previous PCI	64/605 (10.6%)	10/70 (14.3%)	54/535 (10.1%)	0.29
Previous CABG	14/605 (2.3%)	3/70 (4.3%)	11/535 (2.1%)	0.24
eGFR < 60 mL/min	169/605 (27.9%)	24/70 (34.3%)	145/535 (27.1%)	0.20
Initial cardiac arrest	64/608 (10.5%)	21/72 (29.2%)	43/536 (8.0%)	<0.01

Values are expressed as mean (±SD, standard deviation) or n (%). STEMI: ST-elevation myocardial infarction; n: number; BMI: body mass index; COPD: chronic obstructive pulmonary disease; PCI: percutaneous coronary intervention; CABG: coronary artery bypass graft; MI: myocardial infarction.

**Table 2:** Laboratory parameters.

Laboratory Parameters	Cardiogenic Shock (n = 72)	Non-Cardiogenic Shock (n = 536)	*p*-Value
Creatinine in mg/dL	1.27 (1.02, 1.66)	1.10 (0.88, 1.37)	<0.001
Hemoglobin in g/dL	12.35 (11.40, 13.60)	12.85 (11.70, 14.00)	0.061
Platelet count ×1000/UL	218 (173, 277)	216 (178, 271)	>0.9

Values are expressed as mean (interquartile ranges).

**Table 3:** Procedural characteristics.

Characteristics	STEMI Population (n = 608)	Cardiogenic Shock (n = 72, 11.8%)	Non-Cardiogenic Shock (n = 536, 88.2%)	*p*-Value
**Culprit lesion**				
Left main vessel	10/608 (1.6%)	2/72 (2.8%)	8/536 (1.5%)	0.42
Right coronary artery	209/608 (34.4%)	27/72 (37.5%)	182/536 (34.0%)	0.55
Left anterior descending	300/608 (49.3%)	34/72 (47.2%)	266/536 (49.6%)	0.70
Left circumflex artery	77/608 (12.7%)	7/72 (9.7%)	70/536 (13.1%)	0.42
Venous bypass graft	4/608 (0.7%)	0/72 (0.0%)	4/536 (0.7%)	0.46
Arterial bypass graft	4/608 (0.7%)	2/72 (2.8%)	2/536 (0.4%)	0.03
**Procedural data**				
Emergent CAG	593/607 (97.7%)	72/72 (100%)	521/535 (97.4%)	0.30
Femoral artery access	151/608 (24.8%)	30/72 (41.7%)	121/536 (22.6%)	<0.01
Inotropic support	81/608 (13.3%)	55/72 (76.4%)	26/536 (4.9%)	<0.01
Mechanical circulatory support	13/608 (2.1%)	10/72 (13.9%)	3/536 (0.6%)	<0.01
**PCI**				
Failed PCI or CAG-only	60/608 (9.9%)	5/72 (6.9%)	55/536 (10.3%)	0.38
Culprit-only strategy	526/608 (86.5%)	64/72 (88.9%)	462/536 (86.2%)	0.53
MV strategy	22/608 (3.6%)	3/72 (4.2%)	19/536 (3.5%)	0.79
Number of stents	1.28 (0.94)	1.31 (1.01)	1.28 (0.90)	0.82
Contrast volume (mL)	167.2 (79.8)	165.0 (75.9)	167.5 (80.4)	0.81
AKI after procedure	139/474 (29.3%)	20/40 (50.0%)	119/434 (27.4%)	<0.01

Values are expressed as mean (±SD, standard deviation) or n (%). STEMI: ST-elevation myocardial infarction; n: number; CAG: coronary angiography; PCI: percutaneous coronary intervention; MV: multivessel; AKI: contrast-associated acute kidney injury.

**Table 4:** In-hospital outcomes.

Characteristics	STEMI Population (n = 608)	Cardiogenic Shock (n = 72, 11.8%)	Non-Cardiogenic Shock (n = 536, 88.2%)	*p*-Value
Intraprocedural complications	175/608 (28.8%)	33/72 (45.8%)	142/536 (26.5%)	<0.01
Intraprocedural mortality	20/608 (3.3%)	11/72 (15.3%)	9/536 (1.7%)	<0.01
In-hospital mortality	95/608 (15.6%)	32/72 (44.4%)	63/536 (11.8%)	<0.01

Values are expressed as n (%). STEMI: ST-elevation myocardial infarction. n: number.

**Table 5:** Univariate and multivariable Cox regression model to identify independent predictors of death.

Factor	Univariate			Multivariable		
	HR	(95% CI)	*p*-Value	HR	(95% CI)	*p*-Value
Age	1.07	(1.03–1.11)	<0.001	1.08	(1.04–1.11)	<0.001
PAD	1.29	(1.01–1.66)	0.045	1.36	(1.08–1.71)	0.009
Previous MI	2.16	(1.32–3.55)	0.002	1.48	(1.12–1.96)	0.006
Cardiac arrest	1.59	(1.29–1.96)	<0.001	1.66	(1.36–2.02)	<0.001
Female sex	1.07	(0.85–1.35)	0.6			
CKD	1.23	(0.96–1.57)	0.10			
Cardiogenic shock	2.54	(1.84–3.50)	<0.001	2.08	(1.53–2.84)	<0.001
Smoke	0.98	(0.80–1.21)	0.9			
BMI	0.97	(0.93–1.01)	0.15			

PAD: peripheral artery disease; MI: myocardial infarction; CKD: chronic kidney disease.

**Table 6:** Univariate and multivariable regression model to identify independent predictors of cardiogenic shock.

Factor	Univariate			Multivariable		
	OR	(95% CI)	*p*-Value	OR	(95% CI)	*p*-Value
Age	1.05	(0.96–1.14)	0.26			
Female sex	1.61	(0.98–2.67)	0.06	1.49	(0.87–2.53)	0.14
PAD	2.18	(1.30–3.67)	<0.01	2.25	(1.29–3.92)	<0.01
Previous MI	2.26	(1.25–4.08)	<0.01	1.64	(0.87–3.10)	0.13
Cardiac arrest	4.72	(2.60–8.57)	<0.01	4.36	(2.32–8.21)	<0.01
CKD	1.41	(0.83–2.38)	0.21			
Culprit: LAD	0.91	(0.55–1.49)	0.70			

PAD: peripheral artery disease; MI: myocardial injury; CKD: chronic kidney disease; LAD: left anterior descending artery.

## Data Availability

Data are available from the authors upon reasonable request.

## Supplementary Materials

The following supporting information can be downloaded at: https://www.mdpi.com/article/10.3390/jcm14020504/s1, Table S1: In hospital complications; Table S2: Therapy at discharge.
