# Supplementary material for: Predictors and Impact of Cardiogenic Shock in Oldest-Old ST-Elevation Myocardial Infarction Patients
Source: J Clin Med. 2025 Jan 14;14(2):504. doi: 10.3390/jcm14020504 (PMC11766001; doi:10.3390/jcm14020504)
Supplement: Supplementary file 1 [file jcm-14-00504-s001.zip › jcm-3325242-supplementary.pdf]

Supplementary Table S1. In hospital complications.

Complication	STEMI population (n=608)	Cardiogenic Shock (n=72, 11.8%)	Non-Cardiogenic Shock (n=536, 88.2%)	p-value
Ischemic stroke	8 (1)	1 (2)	7 (1)	1.00
Target vessel failure	9 (1)	1 (2)	8 (1)	1.00
Stent thrombosis	9 (1)	2(3)	7 (1)	.652
Relapsing angina	10 (2)	2 (3)	8 (1)	.755
Heart failure	171 (28)	33 (46)	138 (26)	<0.001
Major bleedings (BARC 3 or 5)	19 (3)	2 (3)	17 (2)	.857
Minor bleedings	25 (4)	2 (3)	23 (4)	.543

Values are expressed as n (%).

Supplementary Table S2 Therapy at discharge.

Therapy	STEMI population (n=493)	Cardiogenic Shock (n=29)	Non-Cardiogenic Shock (n=464)	p-value
Statins	452 (92)	24 (83)	428 (92)	.073
ACE-Inhibitor/Angiotensin receptor blocker	312 (63)	14 (48)	298 (64)	.084
Betablockers	354 (72)	15 (52)	339 (73)	.013
Calcium channel blockers	60 (12)	2 (7)	58 (13)	.370
Diuretics	302 (61)	23 (79)	279 (60)	.04
Mineralcorticoid receptor antagonists	145 (29)	9 (31)	136 (29)	.843
Nitrates	42 (9)	1 (3)	41 (9)	.313
Proton pump inhibitors	460 (93)	29 (100)	431 (93)	.428

Values are expressed as n (%).
